# A novel hybrid control framework for frequency regulation in RES and EV-enriched power grids of Delhi power distribution utility

**DOI:** 10.1038/s41598-026-53160-9

**Published:** 2026-06-01

**Authors:** Mrinal Ranjan, Ravi Shankar, Santosh Kumar Gupta, Ashish Ranjan, Md Atiqur Rahman, Hasmat Malik, Vinay Kumar Jadoun

**Affiliations:** 1 Department of Electrical and Electronics Engineering, Gaya College of Engineering, Gaya, Bihar 823003 India; 2 Department of Electrical Engineering, NIT, Patna, Bihar 800005 India; 3 Department of Electrical Engineering, Government Engineering College, Siwan, Bihar 841226 India; 4 Department of Electrical and Electronics Engineering, Nalanda College of Engineering, Chandi, Bihar 803108 India; 5 Department of Science, Technology and Technical Education, Bihar, India; 6 Department of Electrical Power Engineering, Faculty of Electrical Engineering, Universiti Teknologi Malaysia (UTM), Johor Bahru, 81310 Malaysia; 7 Manipal Institute of Technology, Manipal Academy of Higher Education, Manipal, India; 8 Department of Electrical Engineering, Graphic Era (Deemed to be University), Dehradun, 248002 India

**Keywords:** LADRC-FOPIDN-(1 + TD) controller, Electric vehicles, Renewable energy sources, Load frequency control, Quasi opposition arithmetic optimization algorithm, OPAL-RT real-time hardware simulator, Energy science and technology, Engineering

## Abstract

The depletion of fossil fuels and the rising demand for electricity are driving the global shift toward renewable energy sources (RES). The primary goal of incorporating RES into the conventional smart grid is to establish a more ecological and eco-friendly energy system. However, due to their lower system inertia, RESs struggle to effectively respond to fluctuations in load demand. This study investigates how a Dual Loop LADRC-FOPIDN-(1 + TD) controller can improve frequency regulation in a multi-area deregulated electricity system while taking RES’s intermittent nature into account. By integrating LADRC with a cascade controller, the proposed approach delivers improved transient performance over selected benchmark controllers (LADRC, FOPIDN, FOPIDN-(1 + TD)) under the tested scenarios. Furthermore, an enhanced Quasi Opposition Arithmetic Optimization Algorithm (QOAOA) is employed to optimize controller parameters for improved efficiency. Simulation results highlight its strong adaptability to specific uncertainties modeled in this study, such as RES intermittency, load fluctuations, and delay effects, ensuring grid stability. Moreover, this study addresses the use of electric vehicles (EVs) to regulate frequencies in a hybrid power system under the conditions of real-time load demand variations. Plug-in Electric Vehicles (PEVs) are incorporated in every system region as a measure to reduce undesirable transient components on load frequency and power sharing. PEVs absorb unnecessary electrical energy and give it back to the grid when needed, which offers useful grid support, particularly within RES-dominated networks. The proposed controller is tested on a better IEEE-39 bus system with real-time load variation and variability of RES, through data provided by BSES Rajdhani Power Limited (BRPL), Delhi. Lastly, OPAL-RT hardware is used to run a real-time simulation environment, which targets the integration of real hardware with a virtual test environment, to test the effectiveness and the robustness of the controller. Finally, the MATLAB simulation results are compared with OPAL-RT hardware results.

## Introduction

Power systems are undergoing significant transformation, driven by growing environmental sustainability concerns. As a result, many traditional fossil fuel-based plants are being phased out and replaced with renewable energy sources, which are expected to become the dominant power supply under global carbon neutrality goals. In addition to helping to reduce grid congestion and transmission losses, modern power systems that integrate RES contribute to more reliable electricity distribution for consumers^1^. Various researchers^2,3^ investigates the use of a wind turbine-driven self-excited induction generator (SEIG) for standalone wind energy systems operating under nonlinear load conditions. Such configurations are particularly important in areas where access to the grid is limited or unavailable. However, the fluctuation in RES outputs and constantly changing electricity demand negatively impact stability and performance of the power system. A mismatch between the generated power (P_g_) and the instantaneous load demand (P_d_) causes the system frequency to deviate from its nominal value^4^. When generated power (P_g_) falls short of the demand (P_d_), the generator slows down, leading to a drop in system frequency; conversely, if P_g_ exceeds P_d_, the frequency increases. Variations in power transmission capability brought on by disruptions like short circuits or generation losses might worsen system stability. During contingencies, RES are disconnected from the utility grid through power electronic converters, which leads to a reduction in the grid’s overall inertia and raises concerns about maintaining system stability^5^. In addition, RES lack stored kinetic energy that can be quickly deployed to address significant power differences. Renewable energy sources (RESs) are integrated into the grid through power electronic converters, which decouple them from the AC system. As a result, their output becomes less flexible because it is governed by maximum power point tracking. Consequently, these systems do not provide inertial response (IR) and are unable to actively contribute to frequency regulation. Therefore, with high variability in generation and reduced system inertia, enhancing frequency regulation techniques has become essential to ensure reliable load balancing^6^.

Energy storage systems (ESSs) used in smart grids are a useful option to frequency deviations brought about by power supply and demand imbalances^7^. The study in^8^ introduces an integrated control approach that enables Hybrid Energy Storage Systems (HESSs) to contribute to primary frequency regulation, addressing frequency instability issues arising from the large-scale integration of Renewable Energy Sources (RESs) through high-voltage aggregation stations in regional power grids. Plug-in hybrid electric vehicles (PHEVs) can be considered one of the most appropriate battery-based storage technologies because of their wide or broad distribution, inexpensive charging options, less dependence on fossil fuel, and lower greenhouse gas emissions^9^. The efficiency and performance of vehicle-to-grid (V2G) control strategies of grid-connected electric vehicles (EVs) and PHEVs have been studied in many works devoted to the frequency regulation, charging needs, and battery control using state-of-charge (SOC) balancing methods,^10^ and^11^ have been devoted to two-area micro-grids systems. These sophisticated techniques go a long way in reducing inter-line power variations and frequency variations. Moreover, automatic generation control (AGC) is an important tool in the stability of the frequency and scheduled power exchange in real-time grid operations^12^.

The pace at which synchronous generators create alternating current is represented by the power system frequency. Deviations from the nominal frequency can disrupt the grid’s economic efficiency and, in extreme cases, lead to system failure. For example, the biggest blackout in India happened on July 30–31, 2012 and affected the North-Eastern regions of the country. It is considered one of the largest power outages in world history, impacting about 620 million people (nearly half of India’s population at that time). The main cause of the blackout was due to overdrawing of power by certain states, which led to a drop in frequency and triggered a cascading failure^13^. The key goal of AGC in a power system is to modulate generator output whenever there are fluctuations in inter-line power and system frequency. Conventional AGC systems function by adjusting an area’s power output based on the ACE, aiming to bring the ACE as close to zero as possible^14^.

So as to control the frequency, different conventional AGC control strategies have been developed, such as the Proportional-Integral (PI) control method, the Proportional-Integral-Derivative (PID) control method^15^, and its improved method, which are favoured for their reduced settling time and minimal peak overshoot^16^. These methods are the classical control methods, which are easy to tune. However, they cannot adjust the frequency in a large range under different disturbances. These conventional controllers often rely on trial-and-error tuning methods, which can be inefficient and time-consuming^17^.

In order to overcome these drawbacks, studies such as those in^18^ and^19^ have reviewed recent developments in AGC and LFC. These reviews cover a broad spectrum of control techniques, including traditional techniques, adaptive methods, intelligent control systems, model predictive control^20^, optimal control method, fuzzy logic, neural networks, LADRC, and sliding mode control (SMC), Cheetah Optimizer Algorithm (COA^21^ etc.… While these sophisticated control approaches have proved effective in attaining performance objectives, they frequently employ conservative control measures to maintain strong stability and performance. But these controllers might result in sub-optimal control performance, longer response times, and poor resource utilisation. The fast-paced development of smart grids has led to a growing integration of converter-driven technologies, adding new layers of complexity and non-linear behavior to modern power systems. As a result, new control systems have arisen that focus on estimating disturbance inputs^22^.

Due to a strong ability to actively estimate and cancel conflicts (like load changes and variability in renewable energy) in real-time, various researchers discussed the LADRC^23^. Unlike model predictive control, which relies on a prediction model that may have errors, LADRC dynamically compensates for disturbances using an Extended State Observer (ESO). But LADRC required proper tuning. If tuning is incorrect, performance can degrade, leading to instability or poor disturbance rejection^24^.

Therefore, to determine the optimal controller parameters that meet specific design goals, numerous researchers have employed various meta-heuristic optimization algorithms e.g., Imperialist Competitive Algorithm^25^, Salps Swarm Optimization^26^, Archimedes Optimization Algorithm^27^, Wild Horse Optimization^28^, Crow Search Algorithm^29^, Sine Consign Adopted Dingo Optimization Algorithm^30^, Improved Squirrel Search Algorithm^31^, Global Neighbourhood Algorithm^32^, Arithmetic Optimization Algorithm (AOA) etc.^33^. The choice of an optimization technique is influenced by factors such as problem dimensionality, exploration–exploitation balance, constraint handling, convergence rate, and robustness. The superiority of the Arithmetic Optimization Algorithm (AOA) over conventional methods motivated the development of an enhanced version, the Quasi-Opposition-based AOA.

This work contributes to the advancement of LFC research in a following way:

1. Propose a novel Dual Loop LADRC-FOPIDN-(1 + TD) controller that combines various advanced control strategies. The proposed method is better than the conventional control mechanisms, when compared has better frequency regulation performance.
2. The efficacy is demonstrated by a multi-area restructured smart grid system with nonlinear features and potentiality of the proposed controller against the LADRC, FOPIDN and FOPIDN (1 + TD) controllers, considering the fluctuating character of solar energy and wind energy.
3. The tuning of the proposed controller’s parameters has been presented into an improved QOAOA controller. It also has greater merits than other current evolutionary algorithms demonstrated.
4. Examines the effect of EVs on frequency and inter-line power deviances in an intelligent grid system with deregulation taking into account a variety of situations like step loads, variable loads, changing distributed generation and EV-related time delays of 0, 4 and 8 s.
5. To validate the scalability of the controller, a practical instance of the application of IEEE 39-bus structure is used. An effective application of the Dual Loop LADRC-FOPIDN-(1 + TD) proves its capability to maintain a stable frequency and operate successfully in the actual operating conditions.
6. A real time simulation environment which drives real hardware in a virtual test. OPAL-RT hardware is used to make environment to evaluate the effectiveness of the controller robustness.
7. Finally, the MATLAB simulation result is validated with the OPAL-RT hardware results.

The organization of the paper is as follow: the mathematical modelling of the LFC for a multi-zone power system is covered in Sect. “Mathematical modelling of the LFC model for multi-area restructured power system”. The design and assessment of the suggested controller are shown in Sect. “Proposed QOAOA based LADRC-FOPIDN-(1+TD) controller scheme”. Section “Optimization” details the improved QOAOA used to optimize controller gains, along with a comparison highlighting its advantages over other recent evolutionary algorithms. Section “Simulation and experimental results demonstration” deliberates simulations and experimental results from several scenarios. Lastly, Sect. “Conclusion” delivers the overall conclusions of the study.

## Mathematical modelling of the LFC model for multi-area restructured power system

**Fig. 1:**
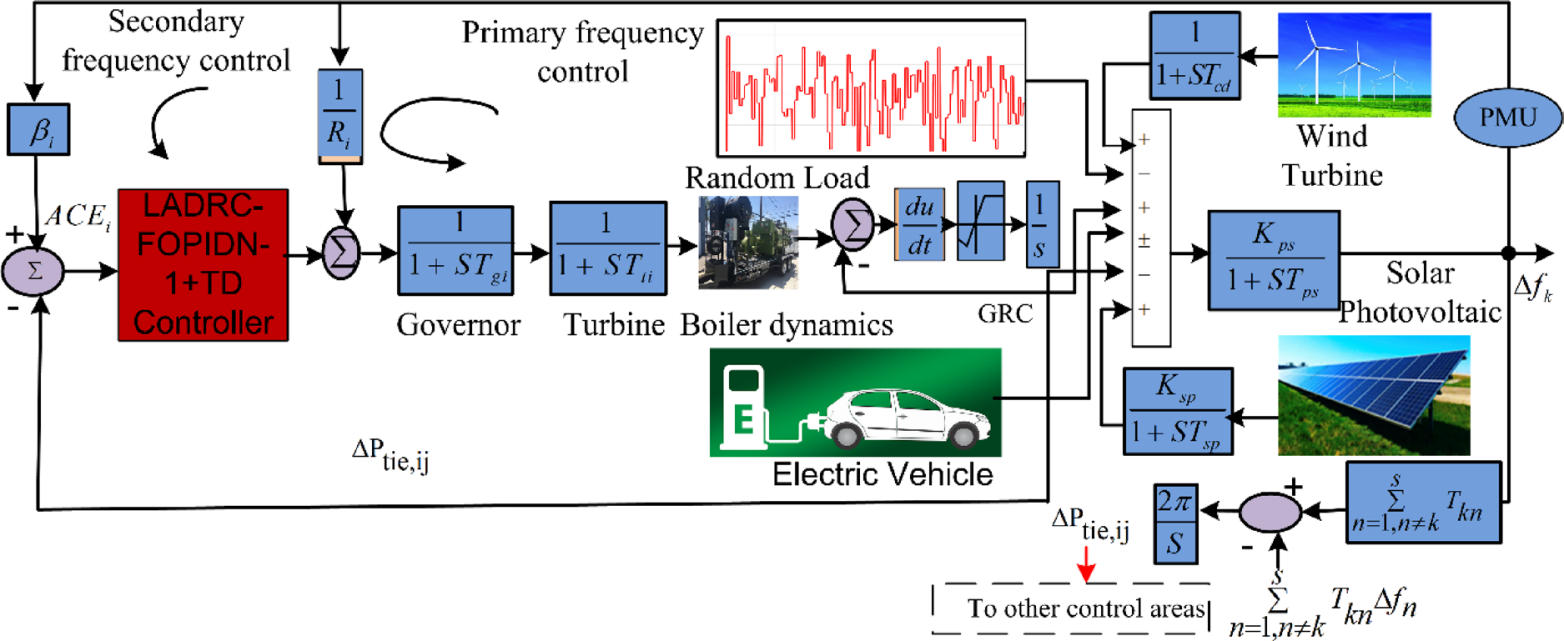
Systematic diagram of ith area LFC model in a smart grid system.

This article aims to examine the efficacy of a projected control strategy and the role of EVs in providing ancillary services for frequency regulation within the context of the LFC issue. To this end, a modified multi-area interrelated smart grid system model incorporating RES and EVs is presented in Fig. 1^34^. The test structure contains of identical control areas, each with a generation capability of 2000 MW. These areas integrate a mix of power sources, including thermal units, biogas plants, and distributed generation units like wind turbine generators (WTG) and SPV systems. The model also accounts for physical limitations like GDB and generation rate constraints (GRC). GRC reflects the limitations in generation speed imposed by practical thermal and mechanical considerations, while GDB represents a range of continuous speed changes that do not result in any valve movement. Details parameters is given in Table 1. The study is conducted in a deregulated power market, incorporating plug-in electric vehicles (PEVs) operating in various modes. Each control area employs a centralized controller, specifically the (LADRC-FOPIDN-(1 + TD)) controller, to effectively minimize the ACE signals. The QOAOA method is engaged to optimally adjust the gain parameters of the suggested controller.

**Table 1:** Key variables defining the proposed power system.

Parameters	Symbols	Values
No of electric vehicles	N_EV_	10,000
Synchronizing coefficient of area n	T_kn_	0.086
Power system’s gain and associated time-constant	Kps,Tps	120,20
Droop constant and frequency bias factor of area i	R_i_, β_i_	2.4, 0.545
Steam governor and turbine time-response constants	T_g_ , T_t_	0.8,0.3
Steam reheats coefficient and time constant	K_r_,T_r_	0.3,10
Biogas unit’s lead-lag phase time constants	X_G_,Y_G_	0.6,1
Valve actuator and discharge delay of bio biogas plant	bv, T_cd_	0.049,0.2
Combustion reaction delay and biogas delay	T_CR_, T_F_	0.01, 0.239
Electric vehicles’ charging time constant and SOC limit	T_*EV*,_ SOC_min_/SOC_max_	0.05, 0.9/0.1
Electric Vehicle ramp rate and Power increase coefficient of EVs	*δ*_*e*_ , *μ*_*e*_	0.05, 0.5

The ith area frequency dynamic equations in the interrelated system structure represented in Fig. 1 can be stated as:

1 $$ACE_{i} { = }\beta_{i} \Delta f_{i} { + }\sum\limits_{j = 1,j \ne i}^{N} {\Delta P_{tie,ij} }$$

Here, *∆f*_*i*_ denotes the frequency deviance within zone-i, while *∆P*_*tie,ij*_ refers to the inter-line power deviation among area-i and area-j. As described in Fig. 1, the LFC model determines the ACE signal for each area-i based on frequency deviance data obtained from Phasor Measurement Units (PMUs). Using this information, it then derives the power adjustment *∆P*_*ci*_ at a given moment according to (2).

2 $$\Delta P_{ci} = T_{p} ACE_{i}$$

In (2), *T*_*p*_ represents the transfer function associated with the LADRC-FOPIDN-(1 + TD) controller. The adjusted ACE control signal is subsequently directed to the governor, which modifies the turbine speed. This allows the resulting change in mechanical power output *∆P*_*mi*_ , to effectively follow the variation in load demand *∆P*_*Li*_. This dynamic adjustment impacts the generator speed, helping to regulate and maintain system frequency at its minimal level. To further analyse the functionality of LFC in power systems, this study examines the state-space representation of LFC mechanism. The state variables chosen for area *i* include *∆P*_*tie,i*_, *∆f*_*i*_, *∆P*_*mi*_, *∆P*_*gi*_, *∆P*_*ci*_. Based on the system illustrated in Fig. 1, the following relationship can be formulated as shown in Eq. 3^35^:

3 $$\left[ \begin{gathered} \Delta \dot{f}_{i} = \frac{1}{{M_{i} }}(\Delta P_{mi} - \Delta P_{Li} - D_{i} \Delta f_{i} - \Delta P_{tie,i} ) \hfill \\ \Delta \dot{P}_{mi} = \frac{1}{{T_{ti} }}(\Delta P_{gi} - \Delta P_{mi} ) \hfill \\ \Delta \dot{P}_{gi} = \frac{1}{{T_{gi} }}(\Delta P_{ci} - \Delta P_{gi} - \frac{1}{{R_{i} }}\Delta f_{i} ) \hfill \\ \Delta \dot{P}_{tie,i} = 2\pi \sum\limits_{j = 1,j \ne i}^{N} {T_{ij} } \Delta f_{i} \hfill \\ \Delta \dot{E}_{i} = \beta_{i} \Delta f_{i} + \sum\limits_{j = 1,j \ne i}^{N} {\Delta P_{tie,ij} } \hfill \\ \end{gathered} \right]\;where\;E_{i} = \int {ACE_{i} }$$

According to the above Equation, the state-space equations for the AGC model of area-i are given by (4) :

4 $$\left\{ \begin{gathered} \dot{x}(t) = Ax(t) + Bu(t) + Dd(t) + w(t) \hfill \\ y(t) = Cx(t) + v(t) \hfill \\ \end{gathered} \right.$$

In the multi-area scheme, the state vector x(t) encompasses key constraints such as frequency deviance (Δ*f*), wind turbine system (WTS) output variation (Δ*P*_*WTS*_), incremental valve point of the governor (Δ*X*_*G*_), the electric vehicles and solar photovoltaic systems ' output changes are Δ*P*_*EV*_ and Δ*P*_*SPV*_. The control input to the microgrid is represented by u(t), while the disruption vector d(t) = [d1(t) d2(t)] consists of wind and SPV fluctuations, namely Δ*P*_*WTS*_ and Δ*P*_*SPV*_. The system’s control responce is represented by *y(t)*. The corresponding inertia of the smart grid is expressed as 2*H*_*t*_, and EV changing aspects are modelled using a time constant *T*_*EV*_. The EV control inputs, Δ*u*_EV_ are constrained by mechanical limitations like as ramp rate *δ*_*e*_, power increase *μ*_*e*_, and the bounds on controllable energy storage, defined by *E*_*max*_ and *E*_*min*_

4a $$A = \left[ {\begin{array}{*{20}c} 0 & {\frac{1}{{2H_{ti} }}} & 0 & {\frac{1}{{2H_{ti} }}} & {\frac{1}{{2H_{ti} }}} \\ 0 & {\frac{ - 1}{{2T_{WTSi} }}} & {\frac{1}{{T_{WTSi} }}} & 0 & 0 \\ {\frac{ - 1}{{RT_{g} }}} & 0 & {\frac{ - 1}{{T_{g} }}} & 0 & 0 \\ 0 & 0 & 0 & {\frac{ - 1}{{T_{EVi} }}} & 0 \\ 0 & 0 & 0 & 0 & {\frac{ - 1}{{T_{SPVi} }}} \\ \end{array} } \right],B = \left[ {\begin{array}{*{20}c} 0 & 0 & 0 \\ 0 & 0 & 0 \\ {\frac{1}{{T_{gi} }}} & 0 & 0 \\ 0 & {\frac{1}{{T_{EVi} }}} & 0 \\ 0 & 0 & {\frac{1}{{T_{SPVi} }}} \\ \end{array} } \right],C = \left[ {\begin{array}{*{20}c} 1 & 0 & 0 & 0 & 0 \\ 0 & 0 & 0 & 1 & 0 \\ 0 & 0 & 0 & 0 & 1 \\ \end{array} } \right],D = \left[ {\begin{array}{*{20}c} {\frac{ - 1}{{2H_{t} }}} & 0 & 0 & 0 & 0 \\ 0 & 0 & 0 & {\frac{ - 1}{{T_{EVi} }}} & 0 \\ \end{array} } \right]^{T}$$

4b $$x(t) = \left[ {\begin{array}{*{20}c} {\Delta f_{i} } & {\Delta P_{WTSi} } & {\Delta X_{Gi} } & {\Delta P_{EVi} } & {\Delta P_{SPVi} } \\ \end{array} } \right]^{T}$$

4c $$u = \left[ {\begin{array}{*{20}c} 0 & {\Delta u_{EVi} } & 0 \\ \end{array} } \right]^{T}$$

4d $$d = \left[ {\begin{array}{*{20}c} {d_{1} } & {d_{2} } \\ \end{array} } \right]^{T}$$

Deregulation allows multiple self-governing participants, including generation companies (GENCOMs), transmission operators, and distribution firms (DISCOMs) to participate in the electricity market. The agreements established between DISCOMs and GENCOMs are captured using a DISCOM Participation Matrix (DPM)^36^. In the system under consideration, it is considered that every zone consists of two GENCOMs and two DISCOMs. The equivalent DPM for this configuration is given as follows:

5 $$DPM = \left[ {\begin{array}{*{20}c} {CPf_{11} } & {CPf_{12} } & {CPf_{13} } & {CPf_{14} } \\ {CPf_{21} } & {CPf_{22} } & {CPf_{23} } & {CPf_{24} } \\ {CPf_{31} } & {CPf_{32} } & {CPf_{33} } & {CPf_{34} } \\ {CPf_{41} } & {CPf_{42} } & {CPf_{43} } & {CPf_{44} } \\ \end{array} } \right]$$

### Solar photovoltaic modelling (SPV)

The integration of renewable energy enhances distributed systems by offering a sustainable solution to support the rising energy requirements of the expanding EV ecosystem^37^. An SPV system uses semiconductor materials, usually silicon, to directly convert sunlight into electricity. This technique greatly aids in the transition to green energy by offering a sustainable, renewable, and ecologically beneficial means of power generation^38^ The system is modelled as a function of solar irradiance, panel efficiency, and surface area. To reflect real-world variability, stochastic fluctuations in solar irradiance are incorporated using a uniformly distributed uncertainty component.

6 $$P_{SPV} = AG\eta \left\{ {1 - 5 \times 10^{ - 3} (T_{a} + 25)} \right\}$$

The power produced (*P*_*SPV*_)​ of a solar photovoltaic (SPV) system is influenced by several factors. These contain the system’s efficiency *η*, typically ranging between 9 and 12%, the panel surface area *A* of the SPV calculated in square meters (m^2^), and the solar irradiance *G*, which is the amount of sunlight collected per unit area, measured in watts per square meter (W/m^2^). Moreover, ambient temperature, represented in degrees Celsius (0^c^), also plays a role in system performance.

7 $$\Psi = 0.5H(t) - 0.3H(t - 10) + 0.3H(t - 17) - 0.3H(t - 35) + \Psi_{n} (t)$$

wherever the uncertain aspect of solar radiation $\Psi _{{n}} (t)$∼ *U* (− 0.1, 0.1).

**Fig. 2:**
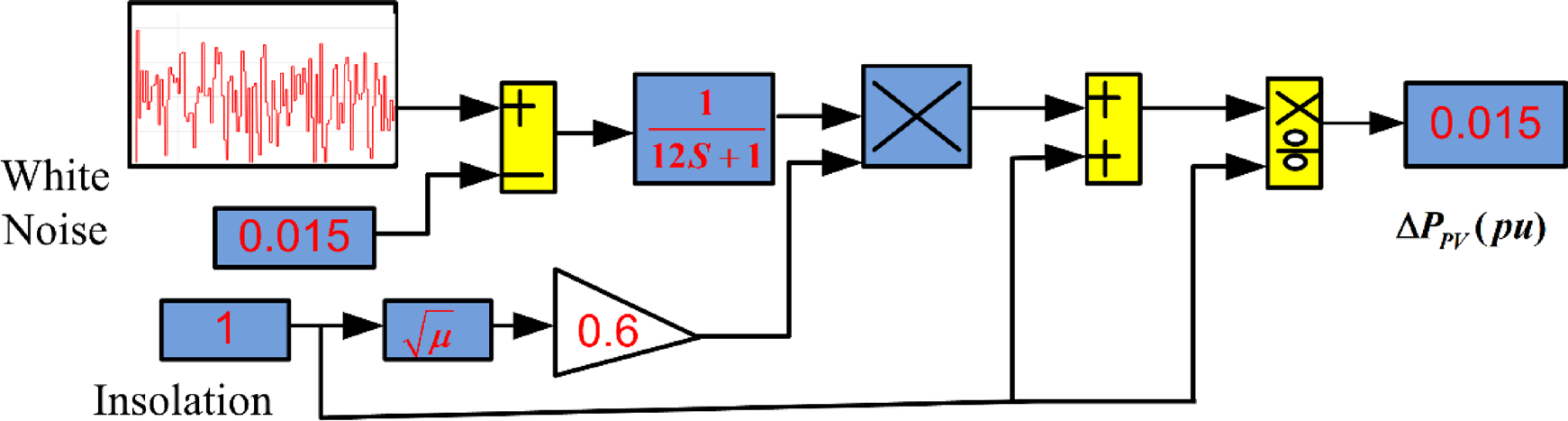
Modelling of solar photovoltaic system.

The intermittency of weather conditions greatly affects the operation of photovoltaic (PV) systems leading to fluctuation in power output in many cases. Such inconsistencies may undermine the stability of the system as it initiates major frequency variations as a result of PV power generation variations. To reduce this challenge, the transfer function that represents oscillations in PV output power that reflects uniform and non-uniform variations in insolation is shown in Fig. 2.

### Wind turbine system modelling (WTS)

The expansion of renewable energy sources (RESs), particularly wind and solar, in both developed and developing nations plays a vital role in ensuring sustainability in the energy sector^39^. Over the past decade, technological advancements have significantly lowered costs, making RES-based energy generation a more attractive option while also contributing to the reduction of greenhouse gas emission. The System is modelled based on aerodynamic principles, where the generated power depends on wind speed, air density, swept area, and power coefficient. The intermittent nature of wind is captured by introducing random variations (white noise components) in wind speed.

The integration of wind energy into smart grid systems familiarizes variability, stemming from the unpredictable and intermittent nature of wind. This variability poses challenges to maintaining system frequency stability. Mechanical power output from the turbine is the product of torque ($T_{WT}$) and rotational speed ($\omega_{mech}$). Whereas its input power ($P_{in}$) is openly linked to the wind speed $V_{wind}$^40^.

8 $$\frac{{d\omega_{mech} }}{dt} = \frac{{T_{WT} - T_{EM,GEN} }}{J}$$

9 $$P_{O} = P_{WT} = T_{WT} \omega_{mech}$$

10 $$P_{in} = P_{wind} = \frac{1}{2}\rho AV^{3}_{wind}$$

11 $$P_{WT} = C_{P} P_{wind} = C_{P} \frac{1}{2}\rho AV^{3}_{wind}$$

12 $$C_{P} = \frac{{P_{WT} }}{{P_{wind} }}$$

13 $$T_{WT} = C_{P} \frac{1}{2}\rho A\frac{{R^{3} }}{{\lambda^{3} }}\omega^{2}_{wind}$$

14 $$\lambda = \frac{{R\omega_{mech} }}{{V_{wind} }}$$

15 $$P_{WT} = C_{P} \frac{1}{2}\rho A\frac{{R^{3} }}{{\lambda^{3} }}\omega^{2}_{wind} \omega_{mech}$$

**Fig. 3:**
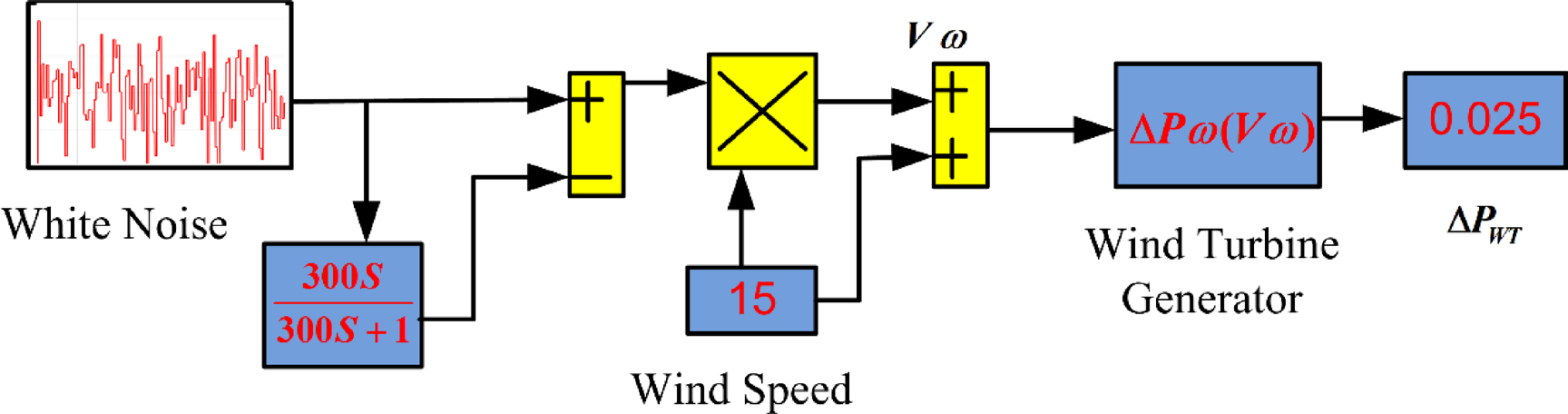
Modelling of wind turbine system.

Here, *ρ* represents the air density (kg/m^3^), while A refers to the swept area of the turbine’s (m^2^). The connection among the turbine power output $P_{WT}$ and the wind power $P_{wind}$ is described in (11). The wind power coefficient, $C_{P}$ introduced in (12), quantifies this relationship. Accordingly, the turbine torque $T_{WT}$ is derived in (13), while the corresponding power output $P_{WT}$ is provided in Eq. 15. Figure 3 illustrates the transfer function-structure of the wind-turbine system. In this transfer-function model, the white noise signal plays a crucial role by introducing a variable speed component, which is subsequently scaled by the predominant wind speed.

### Modelling of plug-in electric vehicles (PEV_S_) for automatic generation control

The EV provides V2G services through bi-directional charging and discharging, enabling frequency regulation. EVs operate as distributed energy storage units within the LFC framework. Their charging/discharging behavior is governed by frequency deviation signals, load demand conditions and SOC constraints. EVs participate in frequency regulation based on SOC levels. For positive frequency deviations, batteries below 50% SOC are charged, while for negative deviations, batteries above 50% SOC discharge power to the grid.

The equivalent circuit model has been widely used because it is intuitive and simple to use, and the charging-discharging characteristics of the battery can be accurately approximated. The charging-discharging behavior of EV batteries is influenced by grid load conditions. When the grid experiences low demand, the batteries undergo charging; conversely, during periods of high demand, the batteries supply power back to the grid. The details mathematical modelling of PEV_S_ and charging and discharging characteristics are developed and presented in a research paper^41^. Communication delays between control center and EV aggregators, response delays in EV charging/discharging systems.

## Proposed QOAOA based LADRC-FOPIDN-(1 + TD) controller scheme

The test system under investigation includes several physical nonlinear limitations, like GDB, GRC, and the integration of variable RESs. Due to these nonlinearities, traditional control methods often struggle to deliver satisfactory performance. Although FOPID controllers offer additional tuning flexibility through fractional orders. they lack explicit disturbance estimation capability. Traditional cascade controllers improve response by separating control objectives but do not inherently address uncertainty and external disturbances.A second-order LADRC has been commonly considered by numerous researchers to handle various uncertainties and disturbances in dynamic systems^23^. It assumes a known linear model structure and uses an Extended State Observer (ESO) to evaluate the system’s internal states along with the overall disturbance, encompassing both model inaccuracies and external influences. But its performance is highly dependent on proper tuning and may suffer from limited transient shaping capability. To overcome these challenges, researchers have introduced a novel QOAOA-tuned LADRC-FOPIDN-(1 + TD) hybrid control strategy and successfully applied it to the studied power system. The subsequent sections detail the design and implementation of this advanced control approach.

### LADRC-FOPIDN-(1 + TD) controller strategy

Unlike traditional ADRC/LADRC or ADRC-PR controllers that operate in a single-loop configuration, the proposed approach introduces a dual-loop control structure.

Consider a standard second-order LADRC model represented by the equation^42^:

16 $$\ddot{P} = bu + f(\dot{P},P,u,d)$$

In Eq. 16, four variables are involved: u, representing the control input; b, denoting the process parameter; f, indicating the total disruption; and d, signifying the external disturbance. A notable advantage of employing LADRC is its ability to function without the necessity of developing a precise mathematical model of the disturbances. Consequently, LADRC remains effective in mitigating or rejecting disturbances even when system modelling is infeasible. The state-space representation considering second-order system, as described in Eq. 16, is presented below.

17 $$\begin{gathered} X_{1} = P \hfill \\ \dot{X}_{1} = X_{2} \hfill \\ \dot{X}_{2} = b_{0} u + X_{3} \hfill \\ \dot{X}_{3} = M \hfill \\ \end{gathered}$$

where $X_{1} = P$ is the output, $X_{2} = \dot{P}$, $X_{3} = f(\dot{P},P,u,d)$, $M = f(\dot{P},P,u,d)$. In the framework of LADRC, the variable $X_{3}$ is familiarized as an comprehensive state to encapsulate the total disturbance *f(t)* affecting the system. This approach enables one to develop an observer which is able to approximate both the internal condition and external disturbances of the system on real time. The overall robustness and performance of the system can be increased by incorporating these estimates into the control law to reduce the effects of disturbances in the system, which is important. This is especially beneficial in cases where the system is difficult or infeasible to obtain a truthful mathematical representation of the system. The observer is characterized as follows:

18 $$\begin{gathered} \dot{V} = A_{0} + B_{0} + L_{0} (P - \hat{P}) \hfill \\ \hat{P} = P_{0} V \hfill \\ \end{gathered}$$

In the outline of LADRC, the system’s output approximation is denoted by $\hat{P}$​, while $b_{0}$​ represents the estimated control gain. This gain is adjusted or calculated based on the plant’s transfer function.

19 $${\mathrm{Where}} A_{0} = \left[ {\begin{array}{*{20}c} 0 & 1 & 0 \\ 0 & 0 & 1 \\ 0 & 0 & 1 \\ \end{array} } \right]\;B_{0} = \left[ {\begin{array}{*{20}c} 0 \\ {b_{0} } \\ 0 \\ \end{array} } \right]C_{0} = \left[ {\begin{array}{*{20}c} 1 & 0 & 0 \\ \end{array} } \right]$$

The state vector is distinct as, $$X = \left[ {\begin{array}{*{20}c} {X_{1} } & {X_{2} } & {X_{3} } \\ \end{array} } \right]^{T}$$ and its estimated complement is $$V = \left[ {\begin{array}{*{20}c} {V_{1} } & {V_{2} } & {V_{3} } \\ \end{array} } \right]^{T}$$. The observer gain vector $$L_{0} = \left[ {\begin{array}{*{20}c} {\beta_{1} } & {\beta_{2} } & {\beta_{3} } \\ \end{array} } \right]^{T} = \left[ {\begin{array}{*{20}c} {3\omega_{0} } & {3\omega_{0}^{2} } & {3\omega_{0}^{3} } \\ \end{array} } \right]^{T}$$ is calculated using the pole-placement method. This method entails choosing the desired observer poles to verify the observer’s stability and enactment. The Estimated State Observer (ESO) can be expressed as follows:

20 $$\begin{gathered} \dot{V}_{1} = V_{2} + \beta_{1} (P - V_{1} ) \hfill \\ \dot{V}_{2} = V_{3} + \beta_{2} (P - V_{1} ) + b_{0} u \hfill \\ \dot{V}_{3} = \beta_{3} (P - V_{1} ) \hfill \\ \end{gathered}$$

In this scenario, the observer approximates the overall disturbance as being three times greater than its initial value. This calculation makes it possible to create a control law that effectively mitigates the disturbance. The control law is expressed as follows:

21 $$u = \frac{1}{{b_{0} }}( - \hat{f} + u_{0} )$$

Consequently, the unique plant is simplified into a much more basic form, as shown below:

22 $$\ddot{P} = (f(\dot{P},P,u,d) - \dot{f}) + u_{0} \approx u_{0}$$

The simplified formulation for $u_{0}$ now incorporates a basic Proportional-Derivative (PD) controller, defined as follows:

23 $$u_{0} = K_{P} (R - V_{1} ) - K_{D} V_{2} - V_{3} ( - \hat{f} + u_{0} )b_{0}$$

**Fig. 4:**
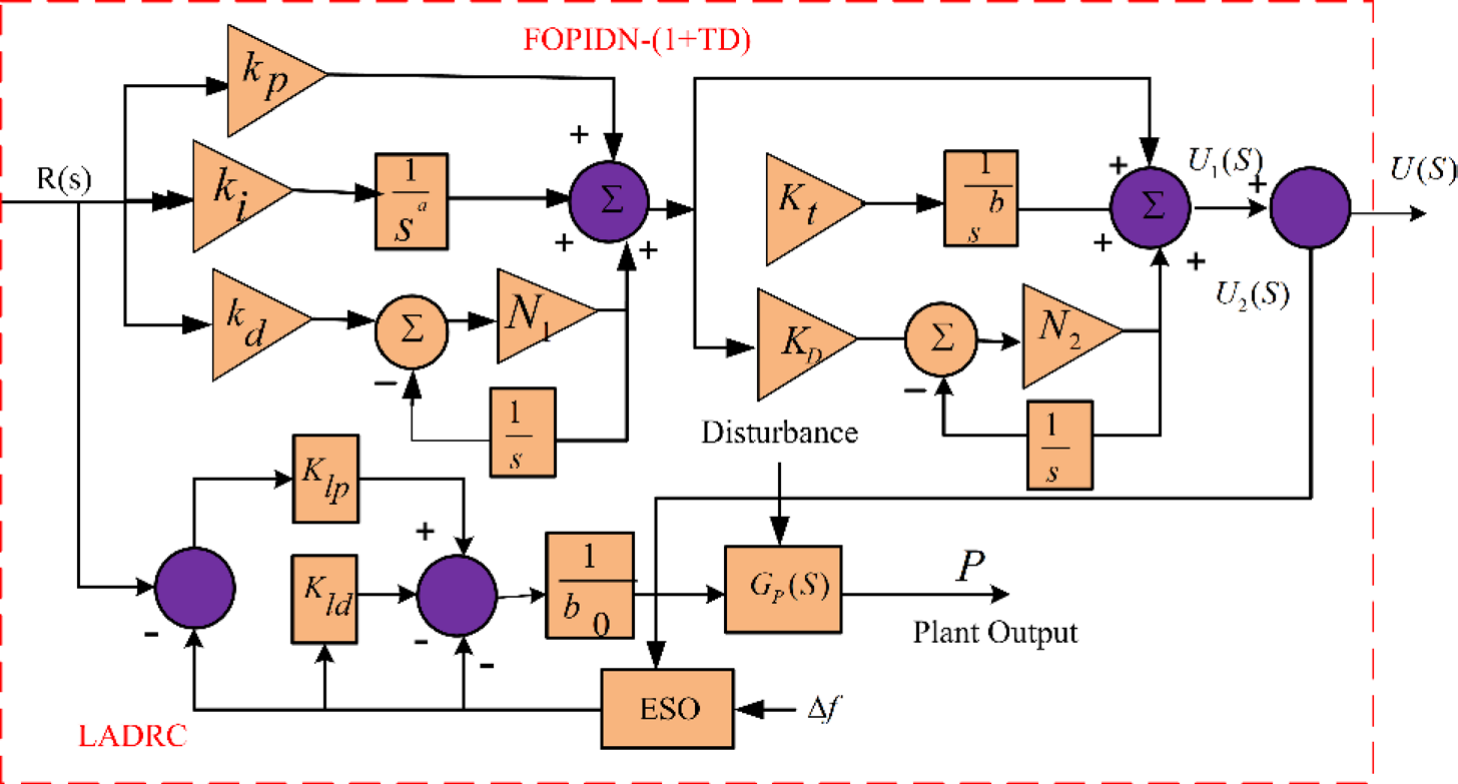
Proposed LADRC-FOPIDN-(1 + TD) controller design.

The control parameters $k_{lp} = \omega_{c}^{2}$ and $k_{ld} = 2\omega_{c}$ in the LADRC correspond to the proportional and derivative gains, correspondingly. The ACE serves as the mention value, denoted as R. The gain of controller and observer are linearly linked to their respective bandwidths, as detailed in^32^. Here $\beta_{1}$,$\beta_{2}$,$\beta_{3}$ are the observer gain, and $\omega_{c}$, $\omega_{0}$ represents the controller and observer bandwidth. The proposed controller takes the ACE defined by Eq. 1. DLFO-LADRC uses two parallel branches The fractional FOPIDN-(1 + TD) branch and LADRC branch whose outputs are summed (Fig. 4 in the revised manuscript).

The overall output u of the proposed controller is equal to the summation of the output response by the cascade controller FOPIDN-(1 + TD) controller u_1_ and the LADRC output response u_2_. The output response of LADRC is given by (24).

24 $$u_{2} = \frac{1}{{b_{0} }}( - \hat{f} + u_{0} )$$

Outer fractional branch transfers FOPIDN-(1 + TD) elements with fractional orders a and b appearing on the integral and derivative terms controller output is specified in (25) as:

25 $$U_{1} (S) = R(S)\left( {k_{p} + \frac{{k_{i} }}{{s^{a} }} + \frac{{N_{1} k_{d} s}}{{s + N_{1} }}} \right)*\left( {1 + \frac{{K_{t} }}{{s^{b} }} + \frac{{N_{2} K_{D} s}}{{s + N_{2} }}} \right)$$

where R(S) represents the ACE signal. Where $s^{a}$ and $s^{b}$ represents the fractional operator and the value of a and b lies in between *0 < a,b łe 1*.Values close to 0 remove the fractional effect; values close to 1 approach integer derivative/integrator behavior. Values > 1 are unusual and increase high-frequency amplification (dangerous for noise). The controller parameters include “$k_{p}$”, “$k_{i}$”, “$k_{d}$”, “$K_{t}$” and $K_{D}$ which stand for the proportional, integral, derivative gains, tilted gain of the FOPIDN-(1 + TD) controller. while “$N_{1}$ ” and $N_{2}$ represents the filter used in the control scheme. The Proposed LADRC-FOPIDN-(1 + TD) controller design is shown in Fig. 4.

## Optimization

### Improved arithmetic optimization algorithm using quasi-opposition-based learning (QOAOA)

**Fig. 5:**
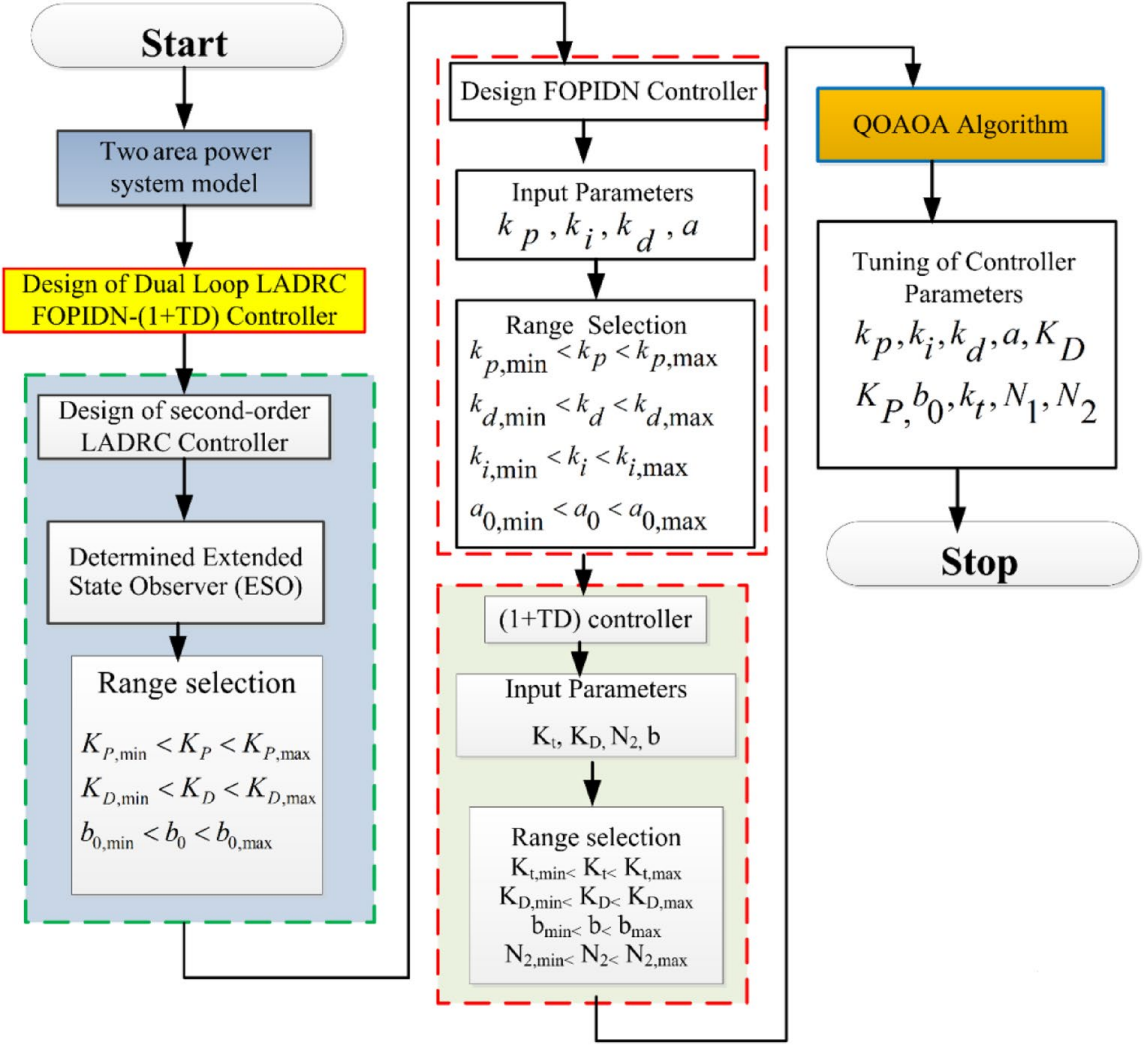
Complete flow-diagram of the improved QOAOA algorithm.

Counter to most of the well-known optimization algorithms, Arithmetic Optimization Algorithm(AOA) has an easy and straightforward implementation, according to its mathematical presentation, to adapt to tackle new optimization problems. Seyedali Mirjalili^43^ proposed the AOA, drawing inspiration from the behavior of arithmetic operations such as division (D), subtraction (S), multiplication (M), and addition (A). It does not need to adjust many parameters except population size and stopping criterion, which are standard parameters in all optimization algorithms. Despite the outstanding performance among several popular algorithms, there is a possibility of being stuck at a local optimal solution, resulting in sluggish behavior. To address this limitation, this research presents an improved variant that integrates Quasi-Opposition-Based Learning (QOBL). By incorporating QOBL, the proposed QOAOA improves both computational performance and reduces the risk of early convergence to local optima. The details stages of improved QOAOA algorithm are illustrated in Fig. 5.

### Quasi opposition-based learning (QOBL)

The present work introduces the concept of quasi-oppositional based learning (QOBL) and its utilization in basic AOA with an intention to increase its convergence speed in order to provide the global optimal solution. Quasi Opposition Arithmetic Optimization Algorithm involves the generation of opposites as well as quasi-opposite values of the initial population as a contrast to only opposite values in the case of opposition-based learning. They integrated to filter out the solutions, moving away from the optimal solution at the initial stage. Thereby the computation speed and chances of not being stuck in local optimal are improved. The main advantage of the Enhanced Arithmetic Optimization Algorithm using Quasi-Opposition-Based Learning (QOAOA) in comparison to other metaheuristic algorithms is its Improved exploration of the search space, reduced risk of premature convergence, Higher accuracy, and consistency across benchmark and real-world optimization problems.

#### Complete pseudocode of QOAOA algorithm

*Step 1*: Randomly generate N individual population $X_{i} \in R^{d} ,i = 1,2,3.....N$ with bound [LB UB].

*Step 2*: Generate the centre of search space $M_{i} = \frac{{Ub_{i} + Lb_{i} }}{2}$.

*Step 3*: Generate the opposite point $X_{Oi} = \left[ {Lb_{i} + Ub_{i} - X_{i} } \right]_{1 \times N}$ where i = 1,2,3,…….N

*Step 4*: Generation of Quasi-opposite point $X_{QOi}$ for N individual population $X_{QOi} = rand(M_{i} ,X_{oi} )$.

If ($$X_{Oj}$$** < **$$M_{j}$$**).**

$$X_{qoj}$$** = **$$M_{j}$$** + (**$$X_{Oj}$$**-**$$M_{j}$$**)***×* r_1_% r_1_ *∈*[0,1].

else.

$$X_{qoj}$$** = **$$X_{Oj}$$** + (**$$M_{j}$$**-**$$X_{Oj}$$**)***×* r_1_.

*Step: 5* Evaluate fitness of both original and quasi opposite solutions.

*Step:* 6 Select the better individual from each pair (original vs. quasi-opposite) to form the updated population.

*Step:* 7 calculate the current finest solution x* in the population.

*Step:* 8 If fitness improved, replaced the previous one and update the global best x* if needed.

*Step:* 9 Return the finest solution x* and its fitness value f(x*).

### Validation of the improved QOAOA algorithm

To estimate the convergence effectiveness of the suggested QOAOA algorithm, the controller gain constraints are improved using five different metaheuristic techniques: PSO, VPL, QOVPL, AOA, and the suggested QOAOA. The above stated optimization algorithm are used to minimalize the objective-function F_ISE_ (Integral of Squared Error (ISE)) defined in Eq. 26 for a multi-area smart network system, while satisfying the various constraints such as k_p,Min_ ≤ k_p_ ≤ k_p,Max_, k_i,Min_ ≤ k_i_ ≤ k_i,Max_, k_d,Min_ ≤ k_d_ ≤ k_d,Max_, a_Min_ ≤ a ≤ a_Max_, K_t,Min_ ≤ K_t_ ≤ K_t,Max_, K_D,Min_ ≤ K_D_ ≤ K_D Max_, b_Min_ ≤ b ≤ b_Max_, N_1,Min_ ≤ N_1_ ≤ N_1,Max_, N_2,Min_ ≤ N_2_ ≤ N_2,Max_, k_lp,Min_ ≤ k_lp_ ≤ k_lp,Max_ , k_lD,Min_ ≤ k_lD_ ≤ k_lD,Max_, b_0Min_ ≤ b_0_ ≤ b_0Max._ Wherever k_p_, k_i_ , k_d_ ,K_D_ , K_t_ are the gain tuning constraints, a, b are the fractional power coefficient,N_1_,N_2_ are the filter coefficient. Each algorithm operates with an initial population of 20 and runs for 10 iterations.

26 $${\mathrm{Minimize}}\;F_{ISE} = \int\limits_{0}^{{T_{sim} }} {\left\{ {\left| {\Delta f_{1} } \right|^{2} + \left| {\Delta f_{2} } \right|^{2} + \left| {\Delta P_{tie} } \right|^{2} } \right\}} dt$$

**Fig. 6:**
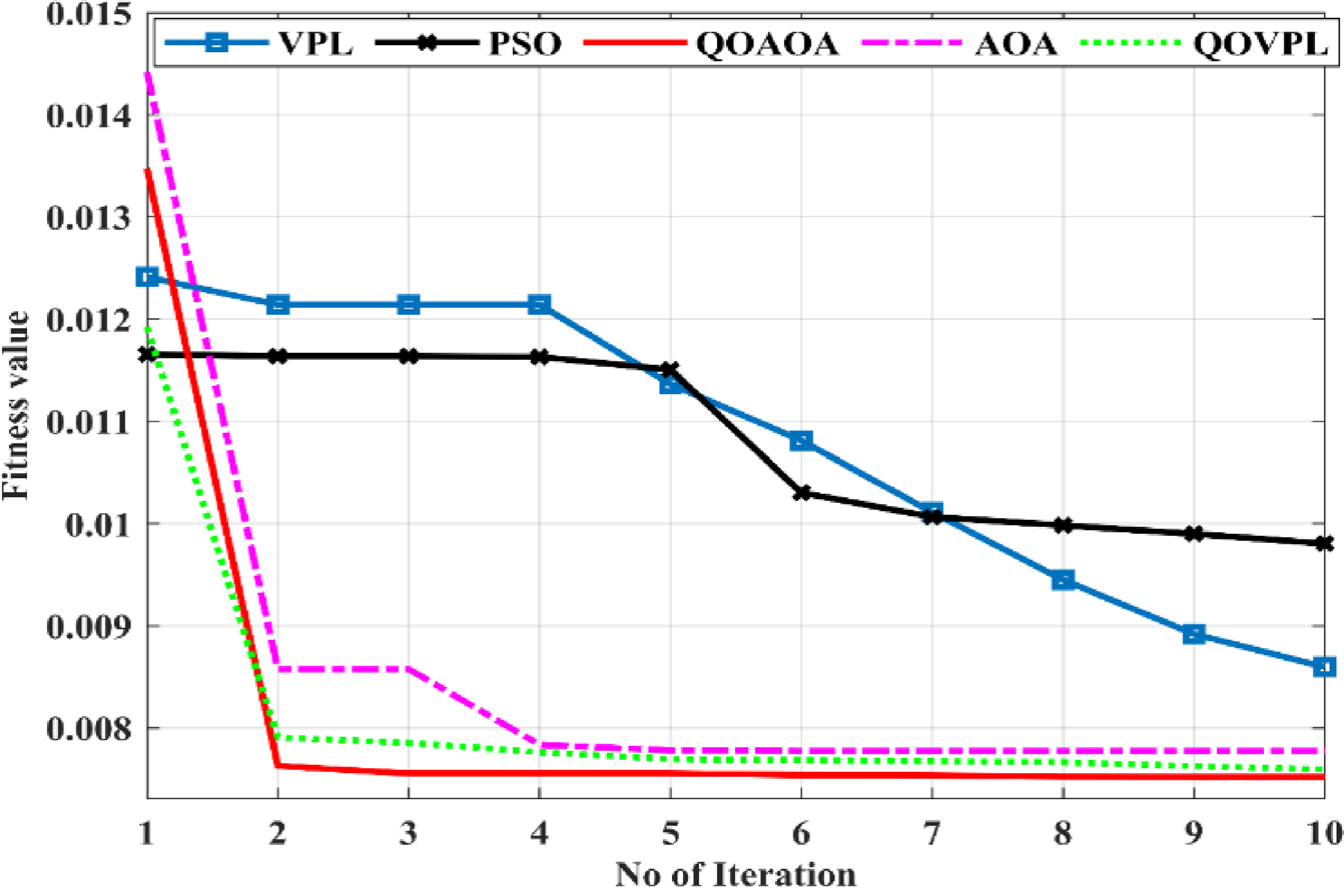
Analysis of different optimization algorithms based on FOD metrics.

All algorithms are applied to the same controller tuning problem under identical conditions, using the Figure of Demerit (FOD) as the objective function. The minimum FOD values obtained using different algorithms are as follows: PSO: 0.0098, VPL: 0.00895, AOA: 0.00798, QOVPL: 0.00489, Proposed QOAOA: 0.00434. Additionally, the convergence characteristics shown in Fig. 6 confirm that QOAOA reaches the optimal solution significantly faster than the other methods, demonstrating improvements in both convergence speed and global exploration capability. These results collectively establish that QOAOA outperforms other algorithms by effectively avoiding local optima and achieving more optimal parameter tuning.

#### Multiple simulation run validation

**Fig. 7:**
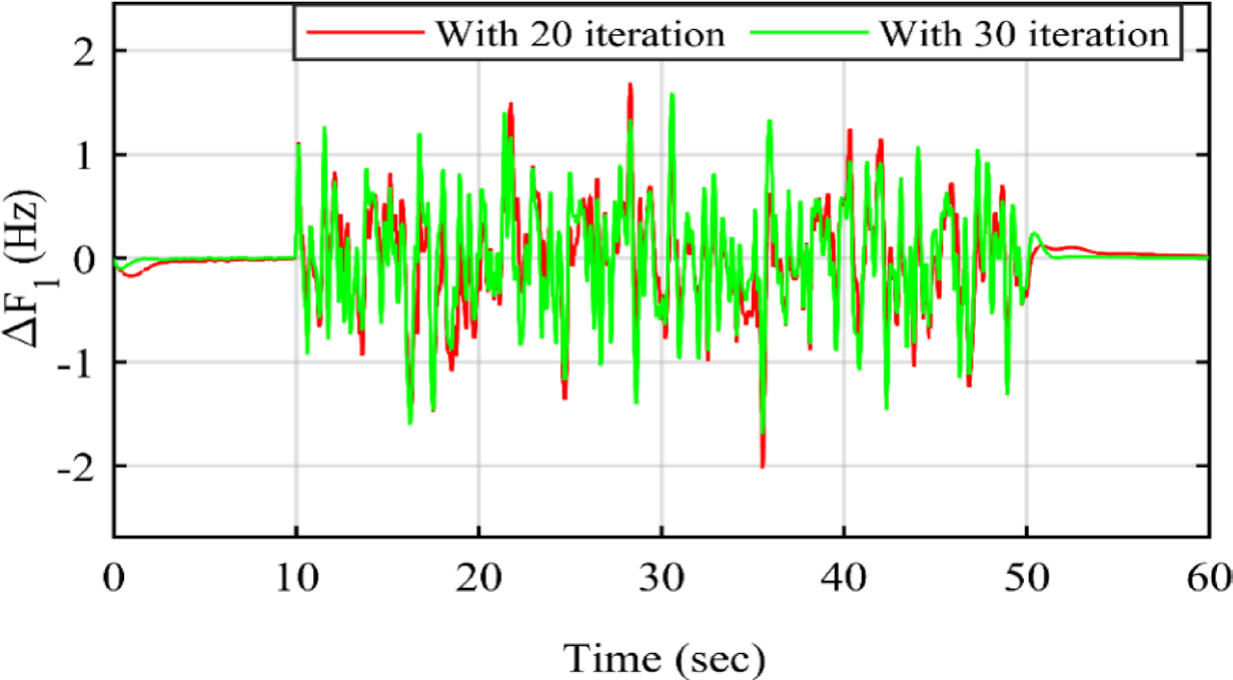
Dynamic response of the system with different no of iteration.

The optimization process has now been executed over multiple independent runs with 20 iterations and 30 iterations to account for the stochastic behavior of the algorithm. The results of dynamic response shown in Fig. 7 demonstrate consistent convergence toward similar optimal solutions, indicating the robustness of the proposed approach.

### Validation of the suggested controller

**Fig. 8:**
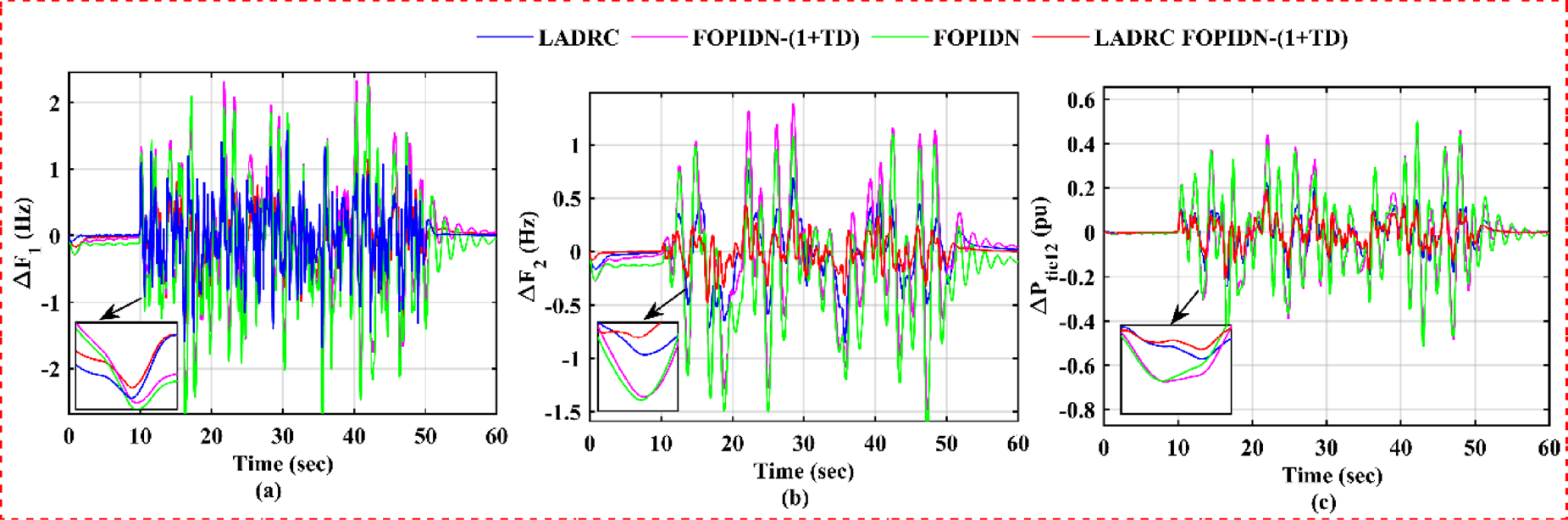
Dynamic performance comparison of various controllers under a 0.1 p.u. sudden load disturbance (SLD) in both areas (**a**) Frequency deviance in area-1, (**b**) Frequency deviance in area-2, and (**d**) Tie-line power discrepancy between area-1 and area-2.

The performance of the recommended controller is evaluated against recent controllers such as LADRC, FOPIDN, FOPIDN-(1 + TD), and LADRC-FOPIDN-(1 + TD) using a two-area smart grid structure in MATLAB/Simulink 2021b. This system accounts for the intermittent characteristics of solar and wind power, along with nonlinearities introduced by the GDB and GRC. The simulation parameters include a time delay (T_d_) of 0.1 s, a GRC of 3% per minute, and a GDB of 0.0006 pu. A novel QOAOA is employed to optimally tune controller parameters. The gain ranges for parameters k_p_, k_i_, k_d_, K_t_, K_D_, k_lp_, k_ld_, a, b are set within [0, 1], while the derivative filter parameters N_1_, N_2_ vary within [0, 10]. The dynamic response of the system under a 0.1 p.u. sudden load disturbance (SLD) in both areas is demonstrated in Fig. 8. Optimal gain values obtained using QOAOA for each controller are presented in Table 2. Table 3 confirms that the projected controller beats the other controller in both transient and steady-state behaviour, with notable enhancements in undershoot and settling time. The enhancement in performance of the present LADRC-FOPIDN-(1 + TD) controller is demonstrated through maximum discrepancy in frequency (Δf_1_, Δf_2_) and inter-line power variation (ΔP_tie,12_) (11.74%, 30.61%) and 36.178% over traditional LADRC and (21.82%,51.57%) and 54.57% over FOPIDN-(1 + TD) controller.

**Table 2:** Optimal gain parameters of various controllers obtained using QOAOA algorithm.

Controllers	Gains	Area-1	Area-2	Controllers	Gains	Area-1	Area-2
Proposed	k_p_	0.9496	0.9481	FOPIDN-(1 + TD)	k_p_	0.9696	0.8889
	k_i_	0.998	0.9423		k_i_	0.9601	0.8352
	k_d_	0.994	0.9650		k_d_	0.994	0.7345
	K_t_	0.936	0.9509		K_t_	0.7016	0.9006
	K_D_	0.975	0.989		K_D_	0.9993	0.8027
	a	0.999	0.989		a	0.7976	0.8505
	b	0.979	0.994		b	0.6852	0.987
	N1	0.965	3.254		N1	6.245	7.454
	N2	0.934	2.125		N2	0.8721	5.214
	K_lp_	0.946	0.9284	FOPIDN	k_p_	0.774	0.9989
	K_ld_	0.9341	0.994		k_i_	0.7233	0.7648
LADRC	K_lp_	0.845	0.912		k_d_	0.9994	0.9995
	K_ld_	0.932	0.985		a	0.8304	0.8294
					N1	1.834	4.2321

**Table 3:** Dynamic behavior comparison of various control strategies.

Peak deviation	Δ F_1_	Δ F_2_	ΔP_tie 12_
Proposed controllers	− 0.7166	− 0.2373	− 0.0785
LADRC	− 0.812	− 0.342	− 0.123
FOPIDN-(1 + TD)	− 0.9167	− 0.490	− 0.1728
FOPIDN	− 1.0097	− 1.1162	− 0.2994
% improvement of proposed controller relative to LADRC	11.74	30.61	36.178
% improvement of proposed controller relative to FOPIDN- (1 + TD)	21.82	51.57	54.57

Hence proposed Dual Loop LADRC-FOPIDN-(1 + TD) controller provides model-independent disturbance rejection through LADRC, offers enhanced flexibility and tuning capability through fractional-order FOPIDN-(1 + TD), ensures robust performance under RES variability and load disturbances, achieves superior transient and steady-state performance, as demonstrated through quantitative comparisons with benchmark controllers. Increasing LADRC bandwidth ω_c_ improves disturbance rejection but may reduce robustness to model uncertainty and amplify sensor noise.

## Simulation and experimental results demonstration

This paper investigates innovative control strategies and the use of electric vehicles (EVs) as ancillary service providers for frequency regulation in the test structure explained in Fig. 1. Electric vehicles (EVs) operate as distributed energy storage units within the LFC framework. Their charging/discharging behavior is governed by frequency deviation signals, load demand conditions and SOC constraints.

To reflect realistic system behavior, the GRC and GDB are set to 0.03 per minute and 0.0006 pu, correspondingly, with non-linearities incorporated into the controller scheme. The research includes a series of detailed analyses, focusing on how EVs influence system stability under dynamic conditions such as fluctuating load demands and the variability of renewable energy sources. Real-world data from BSES Rajdhani Power Limited (BRPL), Delhi, is used to model these situations. A reformed IEEE 39-bus structure serves both as a benchmark and a simulation platform for testing the controller’s effectiveness. In this setup, real-time data for load, SPV, and wind generation are incorporated. To further assess the practicability and real-world performance of the projected control approach, a HIL simulation is performed considering the OPAL-RT OP4510 system.

### Effect of electrical vehicles under renewable energy source variation

#### 1% step-load perturbations in both areas with variable distributed generation (DG)

**Fig. 9:**
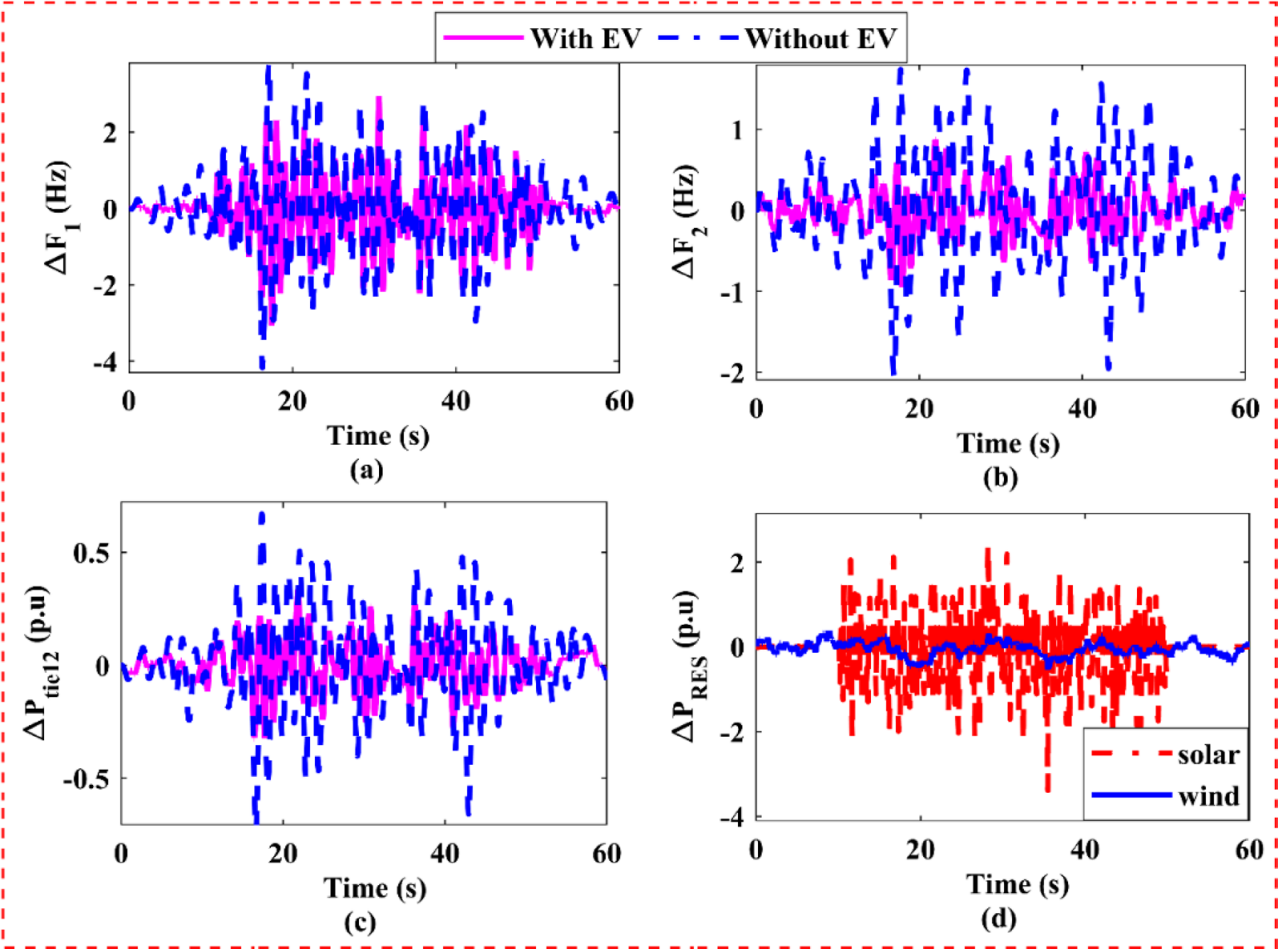
Impact of EV integration on the dynamic system response, (**a**) Frequency deviance in area-1, (**b**) Frequency deviance in area-2, (**c**) Tie-line power deviance between area-1 and area-2, (**d**) Solar and wind power output.

As the controller has proven to be robust against stochastic changes in load, the supplementary support capabilities of EVs are explored in this section. Challenges like the irregular nature of solar photovoltaic (SPV) and wind turbine (WT) outputs, as well as a 1% step-load deviation in both zones, are considered. The variations in SPV and WT outputs are demonstrated in Fig. 9(d). The system dynamic behaviour, measured by frequency and inter-line power fluctuations, is compared for two cases: with and without EV integration, as explained in Fig. 9a–c. Figure 9 clearly demonstrates that the inclusion of electric vehicles (EVs) leads to an enhanced system response. Table 4 presents the maximum deviations in frequency and inter-line power. By integrating electric vehicles (EVs) into the system, performance improvements are achieved, with reductions in maximum frequency deviances ($\Delta f_{1}$,$\Delta f_{2}$) and inter-line power ($\Delta P_{tie12}$) by (36.79%, 69.444%), and 61.733%, respectively.

**Table 4:** Percentage enhancement of frequency response with electric vehicles (EV).

Peak deviation (10^–3^)	Δ F_1_	Δ F_2_	ΔP_tie 12_
Without EV	− 2.8537	− 1.2852	− 0.323
With EV	− 1.8037	− 0.3927	− 0.1236
% improvement with EV	36.79	69.444	61.733

#### 1% step-load disturbances in both areas with incorporating time delay in electric vehicles

**Fig. 10:**
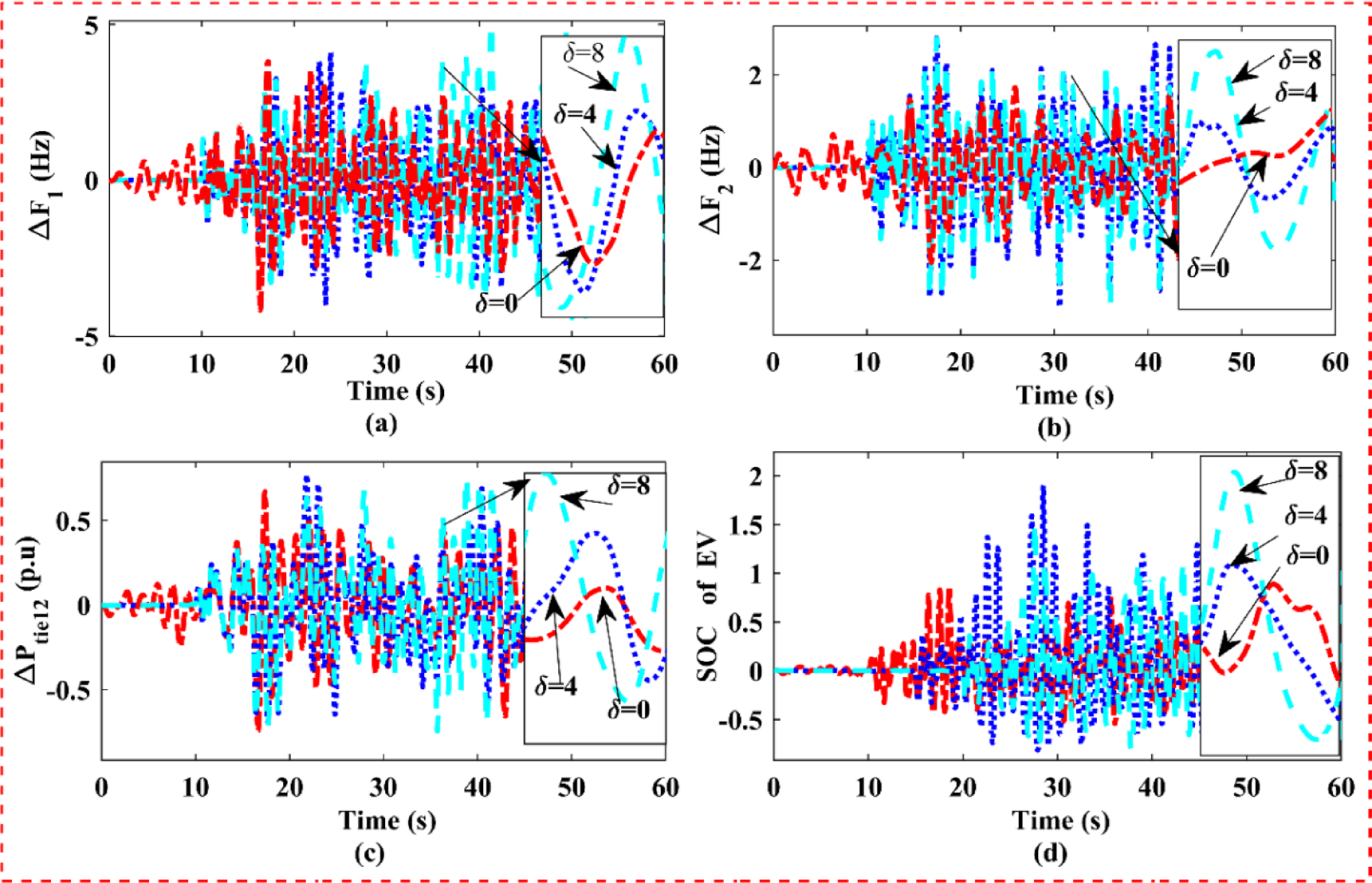
Influence of time delay in EV on system dynamic performance. (**a**) Frequency deviance in area-1, (**b**) Frequency deviancy in area-2, (**c**) Tie-line power deviance between area-1 and area-2, (**d**) SOC graph of EV.

A more rigorous description of time delays has been incorporated, representing communication delays between control center and EV aggregators, response delays in EV charging/discharging systems. The delay is modeled as a transport delay element in the control loop and analysed for different values (e.g., 0 s, 4 s, and 8 s). The effects of communication delays on the effectiveness of EV support are examined in this section. The test system is simulated with time delays of δ = 0, 4 s, and 8 s, while taking RES variability into account. The structure’s dynamic performance under these situations is illustrated in Fig. 10. For this investigation, the load demand is kept constant, represented by a step-load disturbance of 0.01 p.u. As shown in Fig. 10a–c, the frequency and inter-line power deviations increase with longer delay times. This behavior is expected, as delays introduce phase lag and reduce the effectiveness of timely control action. This emphasizes how crucial it is to reduce communication latency while incorporating EVs into the grid. Both area systems use plug-in electric vehicles (PEVs) to help reduce brief disruptions in power sharing and load frequency. Figure 10d illustrates the state-of-charge (SOC) behavior, where PEVs enhance grid stability under high-RES penetration by absorbing excess energy and feeding it back into the grid as needed. From the Fig. 10 it has been observed that by incorporation of time delay degrade system stability, Increase oscillations and settling time, affect synchronization between control actions.

#### Effect of electric vehicles with random load variations in each areas with variable DG

**Fig. 11:**
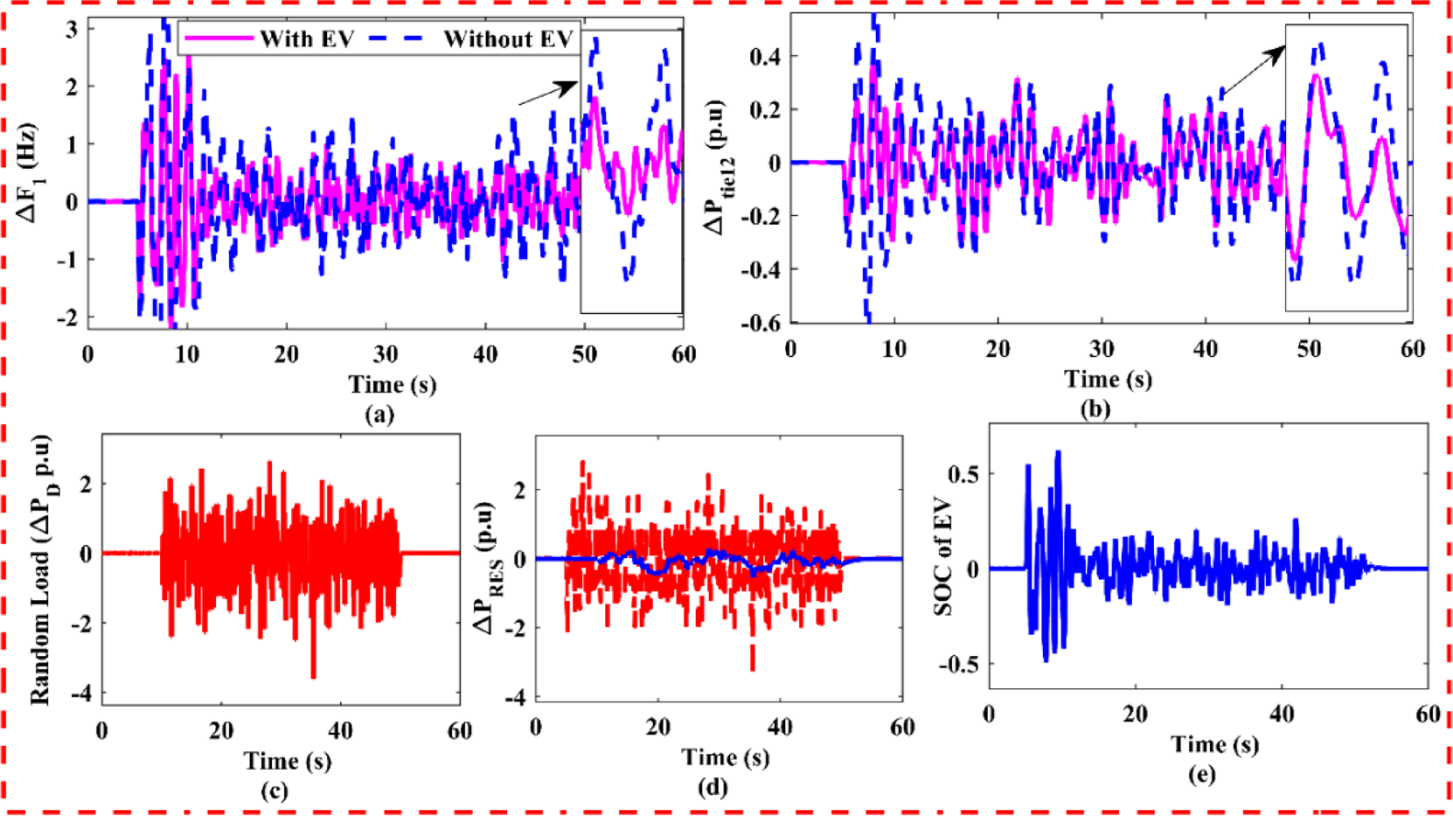
Influence of EV integration on the system’s dynamic response under random load conditions. (**a**) Frequency deviancy in area-1, (**b**) Tie-line power deviance between area-1 and area-2, (**c**) Random load, (**d**) RES output, (**e**) SOC graph.

The problems resulting from the sporadic nature of SPV and wind turbine (WT) outputs, as well as random load variations in each area, are discussed in this section. The load fluctuations are represented in Fig. 11c, while Fig. 11d illustrates the variability in SPV and WT outputs.

The active reaction of the system, focusing on frequency and inter-line power deviations under random-load circumstances, is analysed for two scenarios: with and without EV integration, as shown in Fig. 11a and b. The findings show that frequency overshoot and inter-line power variations are reduced when PEVs are incorporated into the system. Specifically, the incorporation of EVs results in performance improvements of 32.46% in frequency deviance and 30.272% in tie-line power variation. Figure 11e presents the SOC profile, demonstrating the capability of PEVs to store excess energy and supply it back to the grid, when necessary, thereby supporting grid stability in the occurrence of high renewable energy infiltration.

### Controller performance in improved IEEE 39-bus system

**Fig. 12:**
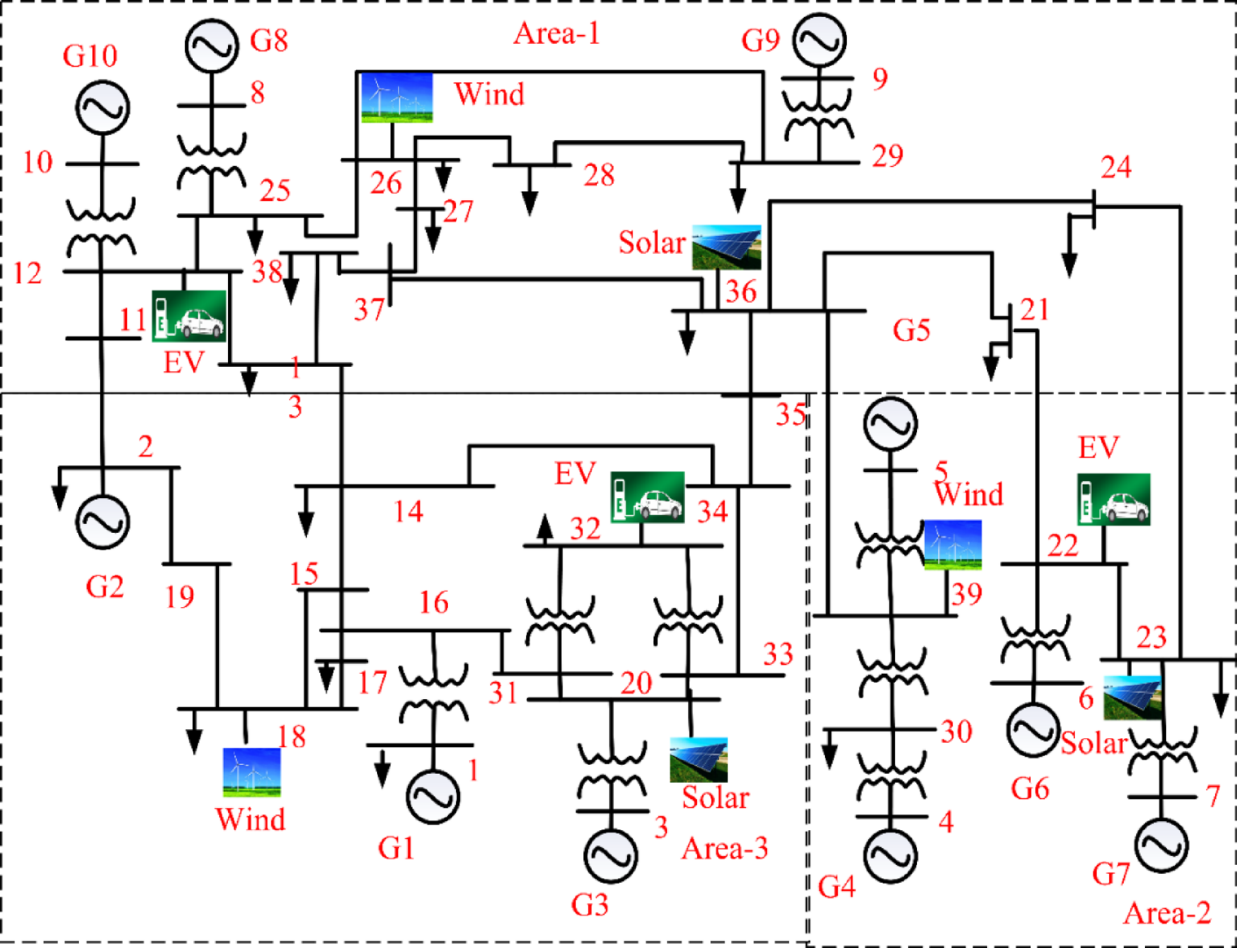
Improved IEEE 39-bus system.

**Fig. 13:**
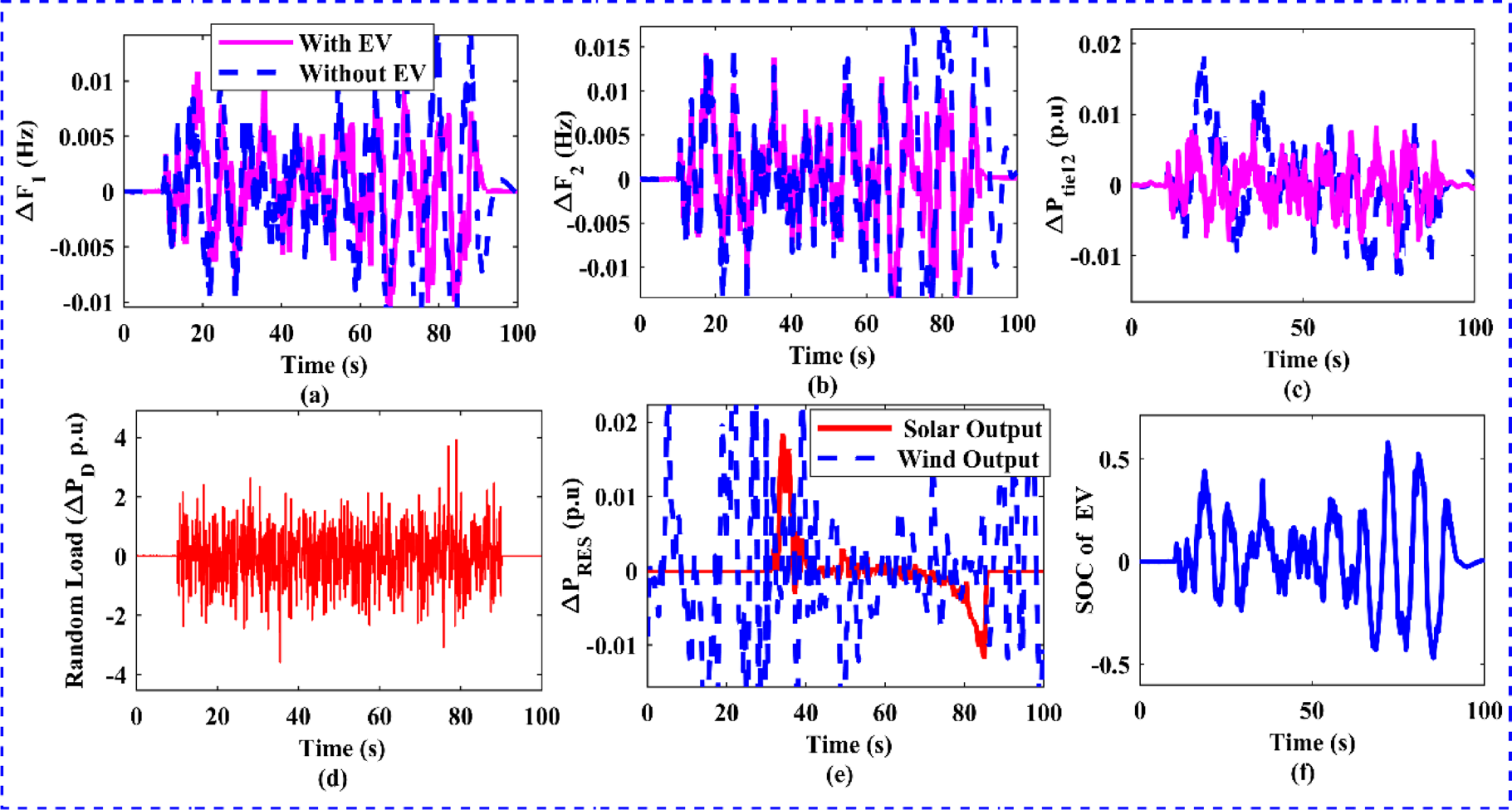
Effect of integration of EV on IEEE 39-bus system (**a**) Frequency deviance in area-1, (**b**) Frequency deviance in Area-2, (**c**) Tie-line power deviance between area-1 and area-2, (**d**) Random load, (**d**) RES output (**e**) SOC graph.

To further demonstrate the effectiveness of the proposed control strategy in a realistic large scale power network, the IEEE 39 bus test system is adopted as the bench mark model. The system consists of 10 Generators, 39 buses, 19 loads, 34 transmission lines and 12 transformers. The IEEE 39 bus system is partitioned into three control areas based on system structure and generator coherency. Generators 3,7, and 9 are selected as representative generators for area-1,2 and 3 respectively. The conventional IEEE 39 bus system is further modified by integrating renewable energy sources and aggregated EV models, variable load patterns, and sudden perturbations as shown in Fig. 12. The scenarios are simulated with real data load demand, solar photovoltaic (PV) power output and wind power obtained at BSES Rajdhani Power Limited (BRPL), Delhi. Figure 13a–c depicts the dynamic performance with and without EV integration of the system, including frequency and inter-line power anomalies of the system with stochastic load conditions as in Fig. 13d. Figure 13e gives the production of the variable load and renewable sources. The frequency deviations associated with the frequency overshoot and inter-line power reductions, as pointed out by the frequency response, are noticeable reduced, by the addition of PEVs. Particularly, EV integration has frequency deviation and tie-line power variation improvements of 45.36 and 23.32 percent, respectively. Figure 13f, demonstrates the SOC characteristics of PEVs, and highlights their role in grid support and energy uptake.

### Experimental test analysis

Traditionally, the confirmation of control systems has been carried out in the field, which can be expensive, inefficient, and potentially dangerous. However, an alternative approach to practical testing is the use of OPAL-RT-based validation, which involves substituting the plant with a corresponding system equipped with inputs and outputs. This method allows for a complete closed-loop investigation, and the OPAL-RT simulator can accurately replicate the plant dynamics, resulting in a significant reduction in risk, cost, and time necessary to test complex systems.

**Fig. 14:**
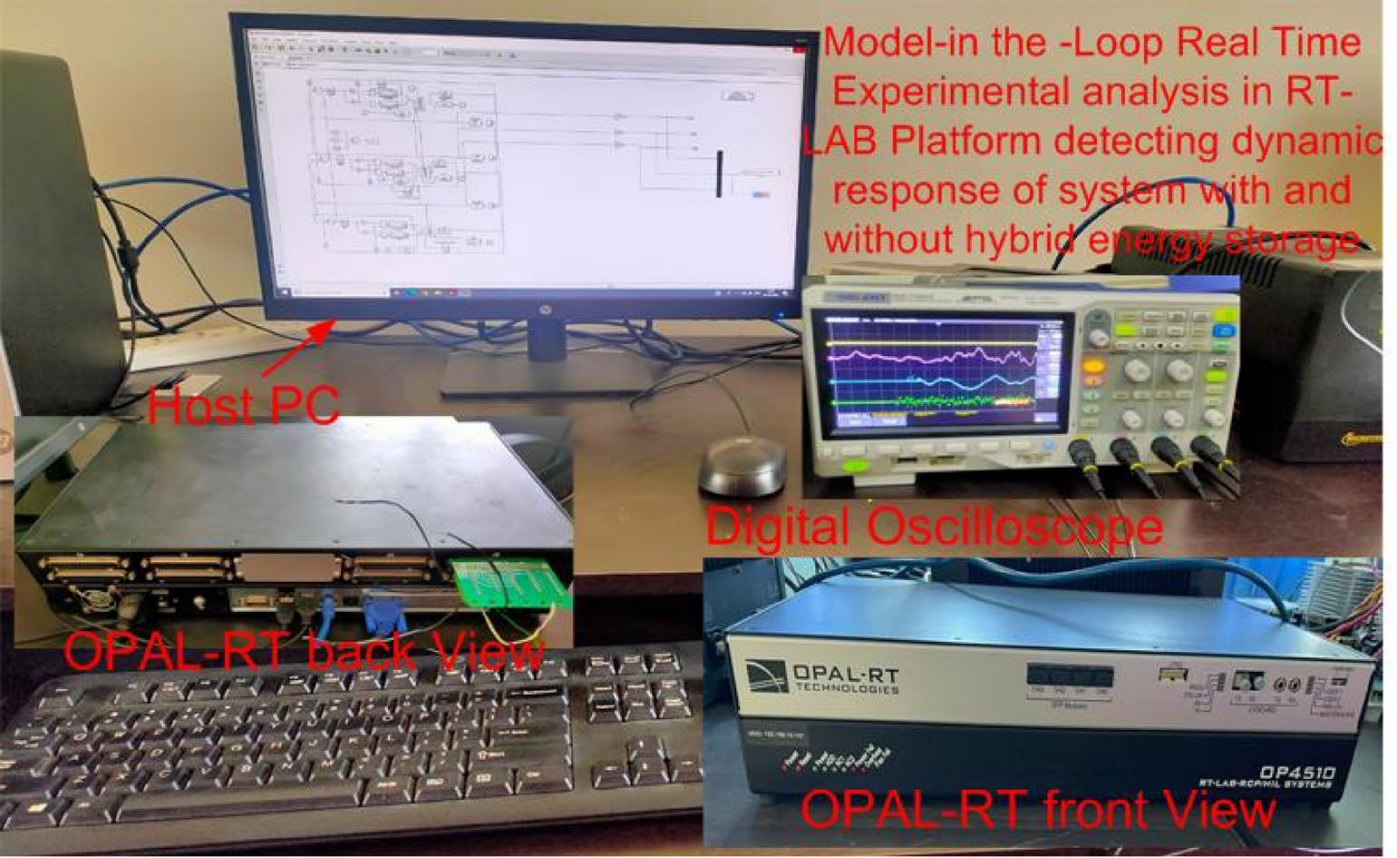
Experimental setup using OPAL-RT simulator.

The OPAL-RT OP4510 platform is used to execute the real-time simulation, and the response is monitored via a digital storage oscilloscope (DSO). The experimental HIL setup, illustrated in Fig. 14, includes^44^:

1. An OPAL-RT Real-Time Simulator (RTS) modelling the system shown in Fig. 1
2. A host PC running MATLAB/Simulink to generate the executable code for the OPAL-RT.
3. A 200 MHz Mixed Signal Oscilloscope to visualize the system waveforms.

**Fig. 15:**
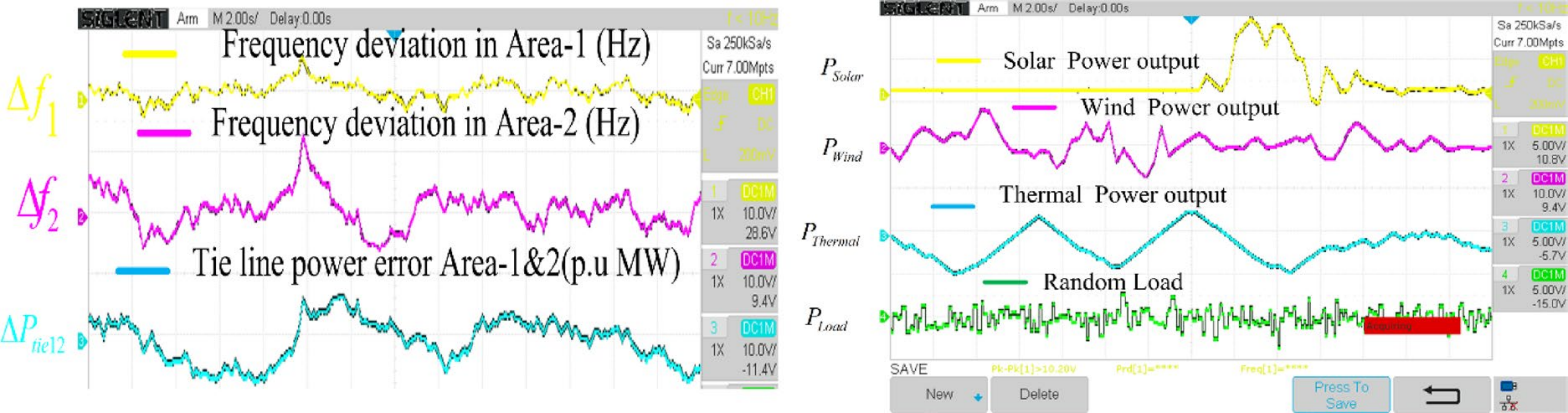
Hardware-in-the-loop OPAL-RT dynamic behavior of the studied system in area-1 and area-2.

The hardware-in-the-loop (HIL) simulator is primarily required to achieve the proposed controller’s accuracy and robustness in a real investigated system. During the real-time study, the sampling time for the proposed system is considered as 50 microseconds. The real-time dynamic response of the investigated system for case 5.1.1 (1% Step-load perturbations in both areas with variable distributed generation is presented in Fig. 14. Dynamic response of the system using OPAL-RT clearly shows that when Electric Vehicles are inserted in the proposed system it reduces the undershoot and settling time. As shown in Fig. 15, the response is smooth and oscillation-free.

#### Validation of simulation results with the OPAL-RT hardware results

A step ahead, in Sect. “Validation of simulation results with the OPAL-RT hardware results” of the revised manuscript, the dynamic response of the investigated system using the proposed controller is compared between MATLAB Simulation and OPAL-RT-based Real-Time Simulator (RTS). To do this, the proposed two areas system with variable DG and EV is first built in MATLAB/Simulink R2021b platform for 60 s of simulation time. The system is then developed via RTLab software integrated with MATLAB for validation. During the real-time study, the sampling time for the proposed system is set to 50 microseconds.

**Fig. 16:**
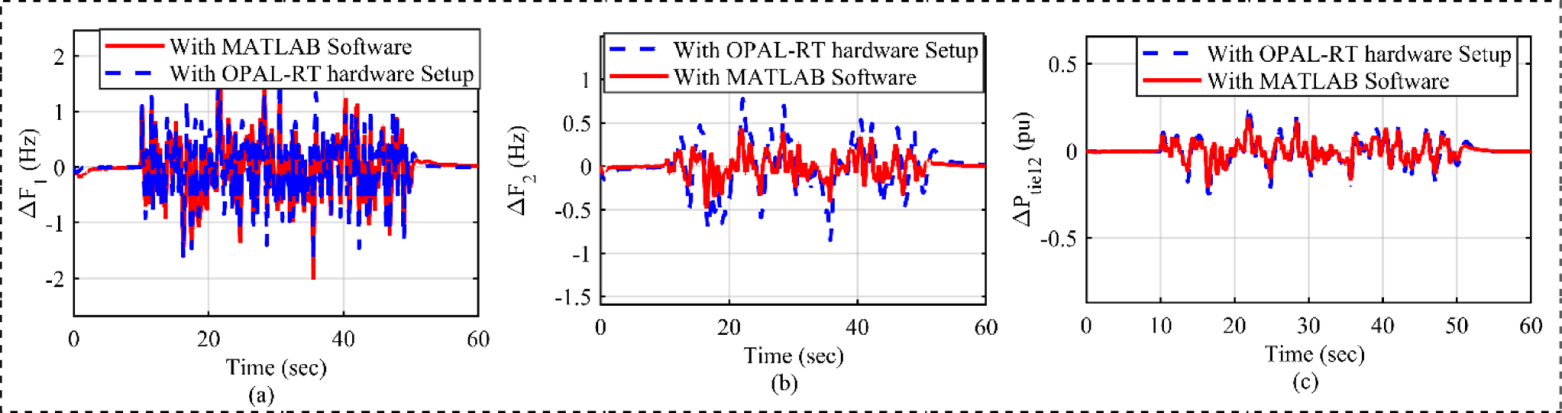
Dynamic response assessment of OPAL-RT and Matlab/Simulink.

Figure 16 illustrates the system’s dynamic response by MATLAB Simulation and OPAL-RT-based Real-Time Simulator (RTS). The critical analysis of the results shows that the system responses from both platforms are almost identical, which validates the proposed system’s practical feasibility using the suggested control strategy.

## Conclusion

To address the challenges in Automatic Generation Control design for smart grids with inherent nonlinearities, this study presents an innovative LADRC-FOPIDN-(1 + TD) control strategy and explores the potential of electric vehicles (EVs) as ancillary service providers for frequency regulation. As demonstrated in Fig. 1, the system incorporates realistic dynamics, with the GRC and GDB set at 0.03 per minute and 0.0006 pu, correspondingly. Nonlinear elements are explicitly integrated into the control design. The investigation thoroughly analyzes the impact of EV participation on system stability under dynamic scenarios such as varying load demand and intermittent renewable energy sources. Real operational data from BSES Rajdhani Power Limited (BRPL), Delhi, is used to create these situations. The controller’s performance is calculated using a modified IEEE 39-bus test system, which incorporates real-time inputs from solar PV, wind, and load profiles. Controller parameters are fine-tuned using a modified QOAOA. Results show enhanced dynamic response, with the proposed controller effectively mitigating specific uncertainties such as RES intermittency, load fluctuations, and delay effects. The study also assesses how different operational modes of PEVs influence frequency control under random load fluctuations. The overall performance of the system is greatly enhanced when PEVs are incorporated into different control regions. To validate the practical applicability of the suggested method, a Hardware-in-the-Loop (HIL) simulation is performed using the OPAL-RT OP4510 platform. Finally, the MATLAB simulation results are compared with OPAL-RT hardware results. In future work further validation under additional operating conditions and advanced controller comparisons can be explored. Future research will also include formal delay margin analysis to precisely quantify the maximum tolerable delay, the proposed controller can handle.

## Data Availability

Data will be provided on request.

## Abbreviations

LADRC: Linear active disturbance rejection control
FOPIDN: Fractional order proportional integral derivative filter
PHEVs: Plug-in hybrid electric vehicles
PSO: Particle swarm optimization
QOVPL: Quasi opposition volleyball premier league
AGC: Automatic generation control
GDB: Governor dead band
SPV: Solar photovoltaic
1 + TD: One plus tilted derivative
EV_S_: Electric vehicles
RES: Renewable energy sources
VPL: Volleyball premier league
SLD: Step load disturbance
LFC: Load frequency control
ACE: Area control error
HIL: Hardware in loop
